# Functional Polymorphisms in *COX-2* Gene Are Correlated with the Risk of Oral Cancer

**DOI:** 10.1155/2015/580652

**Published:** 2015-04-21

**Authors:** Dong Li, Shu-Hong Hao, Yan Sun, Chun-Mei Hu, Zhi-Hua Ma, Zhi Ming Wang, Jie Liu, Hong Bo Liu, Ming Ye, Yu Fei Zhang, Dong Sheng Yang, Guang Shi

**Affiliations:** ^1^Department of Obstetrics and Gynecology, The Second Hospital of Jilin University, Changchun 130041, China; ^2^The Department of Hematology and Oncology, The Second Hospital of Jilin University, Changchun 130041, China; ^3^Department of Oral Surgery, Shengjing Hospital of Youyi Medical University, Jiulong 999077, Hong Kong; ^4^Experimental Technology Center of Youyi Medical University, Jiulong 999077, Hong Kong; ^5^Department of Statistics, School of Public Health, Youyi Medical University, Jiulong 999077, Hong Kong

## Abstract

*Background*. This meta-analysis investigated the association between functional* COX-2* gene polymorphisms and the risk of oral cancer.* Methods*. Several electronic databases were searched for published studies using combinations of keywords related to* COX-2* gene polymorphisms and oral cancer. After selection of relevant studies, following strict inclusion and exclusion criteria, data was performed using STATA 12.0 software.* Results*. We retrieved 83 studies from database search using specific search terms. After multiple rounds of selection and elimination, 7 studies were finally identified as suitable to be included in our present meta-analysis, based on their relevance and data integrity. These 7 studies contained a combined total of 2,296 oral cancer patients and 3,647 healthy controls. Our findings demonstrated that +837 T > C (rs5275) polymorphism in* COX-2* showed statistically significant differences in gene frequencies in case and control groups in allele model and dominant model. Similar results were obtained with* COX-2* gene polymorphism 765 G > C (rs20417). On the other hand, 1195 A > G (rs689466) polymorphism in* COX-2* did not confer susceptibility to oral cancers.* Conclusion*. Based on our results,* COX-2* gene polymorphisms, +837 T > C (rs5275) and −765G > C (rs20417), showed clear links with oral cancer susceptibility, and the 1195A > G (rs689466) polymorphism did not show such a correlation.

## 1. Introduction

Oral cancer is the eighth most common head and neck cancer worldwide with high morbidity and mortality, and an estimated 27,450 new cases and 5,490 deaths were reported in the United States in 2013 [1]. In recent years, increasing incidence of oral cancers, especially in younger age groups, has posed a serious threat to public health [2]. In most countries, oral cancer is more frequent in men than women because of the more prevalent risky habits in men such as alcohol consumption, cigarette smoking, and betel quid chewing [3, 4]. Oral cancers are aggressive and frequently invade as well as metastasize to distant organs, thus making them difficult to cure [5]. The etiology of oral cancer is multifactorial and includes genetic components, environmental components, viral infections, and social and behavioral factors [6]. Individual variations in susceptibility to tobacco-related oral squamous cell carcinoma have been attributed to complex interactions between genetic and environmental factors [7], but the underlying mechanisms appear to converge on inflammation related pathways. Inflammation is closely related to altered gene expression of oncogenes and tumor suppressor genes and is a major factor in promoting neoplastic transformation [8]. Previous studies have established a connection between oral cancers and genetic polymorphisms in* cyclooxygenase* (*COX*), an enzyme that promotes the rate-limiting in the formation of inflammatory prostaglandins [2, 9].

COX-2, also named prostaglandin-endoperoxide synthase (PTGS), is a key enzyme in the arachidonic acid pathway, initiating the synthesis of biologically important prostanoids and eicosanoids [10]. Overexpression of* COX-2* is observed in many cancers, especially in the upper aerodigestive tract cancers, such as oral cancer, gastric cancer, and esophageal cancer, and is associated with cell proliferation, inhibition of apoptosis, tumor invasion, and angiogenesis [11]. The human* COX-2* gene is located on chromosome 1 locus of 1q25.2–q25.3 and is 8.0 kbp in size with 10 exons [12].* COX-2* gene polymorphisms affect the expression levels and enzymatic activity of COX-2 and therefore are intimately linked to inflammatory response and individual variations in the susceptibility to oral cancers [13–15]. Three single-nucleotide polymorphisms (SNPs) in the* COX-2* gene, −1195 G > A (reference SNP ID, rs689466), +837 T > C (rs5275), and −765 G > C (rs20417) have received considerable attention for their close links to oral cancers, compared to the other SNPs of* COX-2* gene [16]. The +837 T > C (rs5275) polymorphism creates an E2F binding site in the promoter to alter* COX-2* expression, and the −765 G > C (rs20417) polymorphism is located in the 3′UTR and influences COX-2 mRNA stability and translation. Both polymorphisms are independently associated with several cancers and evidence shows that polymorphisms of* COX-2* gene, in general, enhance cancer risk [14]. However, controversy exists about the exact role of* COX-2* genetic polymorphisms in cancers, especially in different ethnic groups [7]. Therefore, we conducted this meta-analysis using data extracted from selected case-control studies to investigate the association of three prominent* COX-2* gene polymorphisms with the risk of oral cancer.

## 2. Methods and Materials

### 2.1. Identification of Eligible Studies

To identify all relevant studies on* COX-2* gene polymorphisms and susceptibility to oral cancers, we exhaustively searched PubMed, EBSCO, Ovid, Springerlink, Wiley, Web of Science, VIP, Wanfang, and China National Knowledge Infrastructure (CNKI) databases (last updated search in October, 2014), utilizing selected keywords related to oral Cancer,* COX-2*, and polymorphism genetic. The search terms applied in our literature search were as follows: “Mouth Neoplasms” or “Oral Cancer” or “Tongue Neoplasms” or “Gingival Neoplasms” or “Lip Neoplasms” or “Palatal Neoplasms” or “Salivary Gland Neoplasms” and “Cyclooxygenase 2” or “COX-2” and “Polymorphism, Genetic”. We also manually examined the bibliographies of relevant articles to identify additional studies.

### 2.2. Selection Criteria

The following criteria were applied for literature selection to be included in the present meta-analysis: (1) study types should be case-control studies; (2) the study topic should be related to* COX-2* gene polymorphism and oral cancer susceptibility; (3) the outcome index involved the allele gene and frequencies of genotype in both case and control groups; (4) when the same author published articles using the same clinical data, study with the largest sample size or the newest publication was used; (5) only studies published in English and Chinese are included. The major exclusion criteria were as follows: (1) containing summary and abstracts only; (2) nonhuman studies; (3) duplicate publications or unpublished studies; (4) no sufficient information provided.

### 2.3. Data Extraction and Quality Assessment

The data were extracted from each included study by two independent investigators, and the following information was collected: surname and initials of the first author, year of submission, country, ethnicity, language, age, gender, sample size, genotyping methods, study design, Hardy-Weinberg equilibrium test (HWE), SNP, and gene. Disagreement on the inclusion of any study was resolved by consultation with a third investigator. The quality of included trials was assessed utilizing the critical appraisal skills program (CASP) for case-control Studies (http://www.casp-uk.net/). The CASP criteria are scored as follows: the focused issue is clearly addressed (CASP01); the research problem is eligible and the research design answers the research problem (CASP02); the cases were enrolled acceptably (CASP03); the controls were selected acceptably (CASP04); the measurement for exposure factors is precise to minimize bias (CASP05); the study controls other crucial confounding factors (CASP06); the research results are complete (CASP07); the research results are precise (CASP08); the research results are reliable (CASP09); the research results are applicable to the local population (CASP10); the research results fit with other available evidence (CASP11).

### 2.4. Statistical Analysis

All statistical tests for this meta-analysis were performed with STATA 12.0 (Stata Corporation, College Station, TX, USA). The relative risk (RR) and 95% confidence intervals (CI) were estimated by the fixed effects model or random effects model to evaluate the correlation between* COX-2* gene polymorphism and the risk of oral cancer. *Z* test was applied to estimate the significance of the overall effect size [17]. We used Cochran's* Q*-statistic (*P*_*h*_ < 0.05 was considered significant) and *I* ^2^ tests to quantify heterogeneity among studies [18]. When *P* < 0.05 or *I* ^2^ > 50% indicated heterogeneity random effects model was used; otherwise fixed effects model was employed. In order to reflect the influence of single studies on the results the sensitivity analysis was employed. In addition, potential publication bias was examined by using funnel plots as well as Egger's linear regression test to ensure the reliability of results (*P* < 0.05 was considered significant) [19, 20].

## 3. Results

### 3.1. Study Characteristics

**Figure 1:**
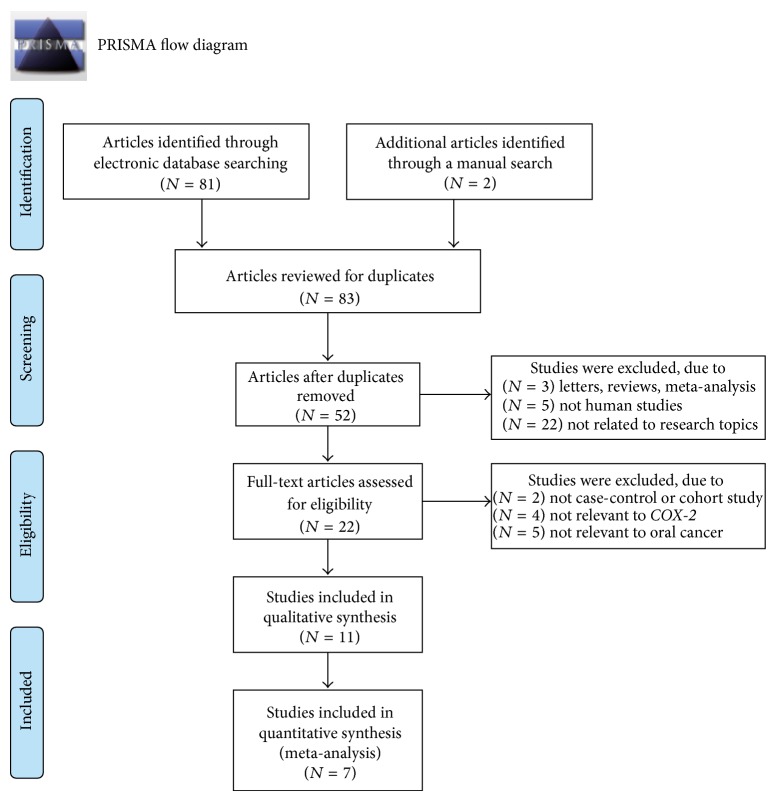
Flow chart shows study selection procedure. Seven studies were included in this meta-analysis.

**Figure 2:**
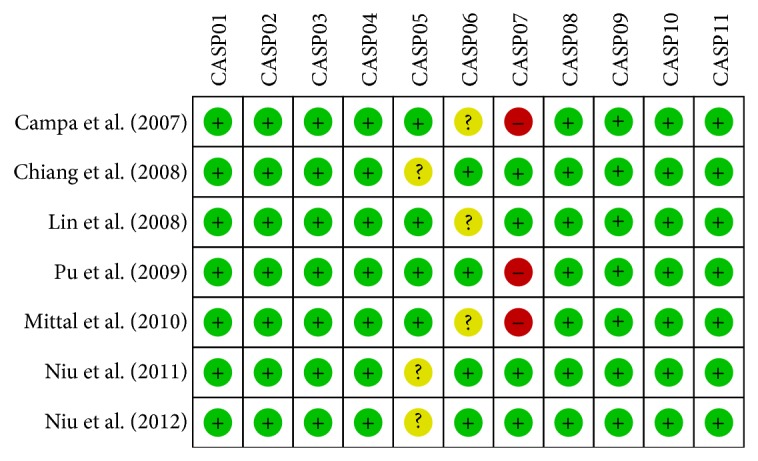
CASP scores for 7 eligible studies for the relationship between functional* COX-2* gene polymorphisms and susceptibility to oral cancer.

A total of 83 articles were retrieved through electronic database and manual searches. After rejecting 31 duplicate studies, 3 letters and reviews, 5 nonhuman studies, and 22 unrelated studies, the remaining studies were reviewed in full text for data integrity. This resulted in further elimination of 11 articles, along with 4 studies that lacked sufficient data (Figure 1). Eventually, 7 clinical studies [7, 21–26], containing a total of 2,296 oral cancer patients and 3,647 control subjects, met our inclusion criteria for quantitative data analysis. These studies were published between 2007 and 2012. Overall, 2 of the seven studies were performed in Caucasians and 5 studies were in Asians (four Chinese and one Indian). Among the 7 included studies, 3 studies used TaqMan method and four studies used PCR-RFLP to detect SNPs, and the patient numbers ranged from 194~1200. Except for +837 T > C (rs5275) allele in the study of Mittal M, 2010 (*P* = 0.006), other genotype distributions were in accordance with HWE (*P* > 0.05). CASP scores and baseline characteristics for eligible studies are presented in Figure 2 and Table 1, respectively.

### 3.2. Meta-Analysis of Association between +837 T > C (rs5275) and the Susceptibility of Oral Cancer

**Figure 3:**
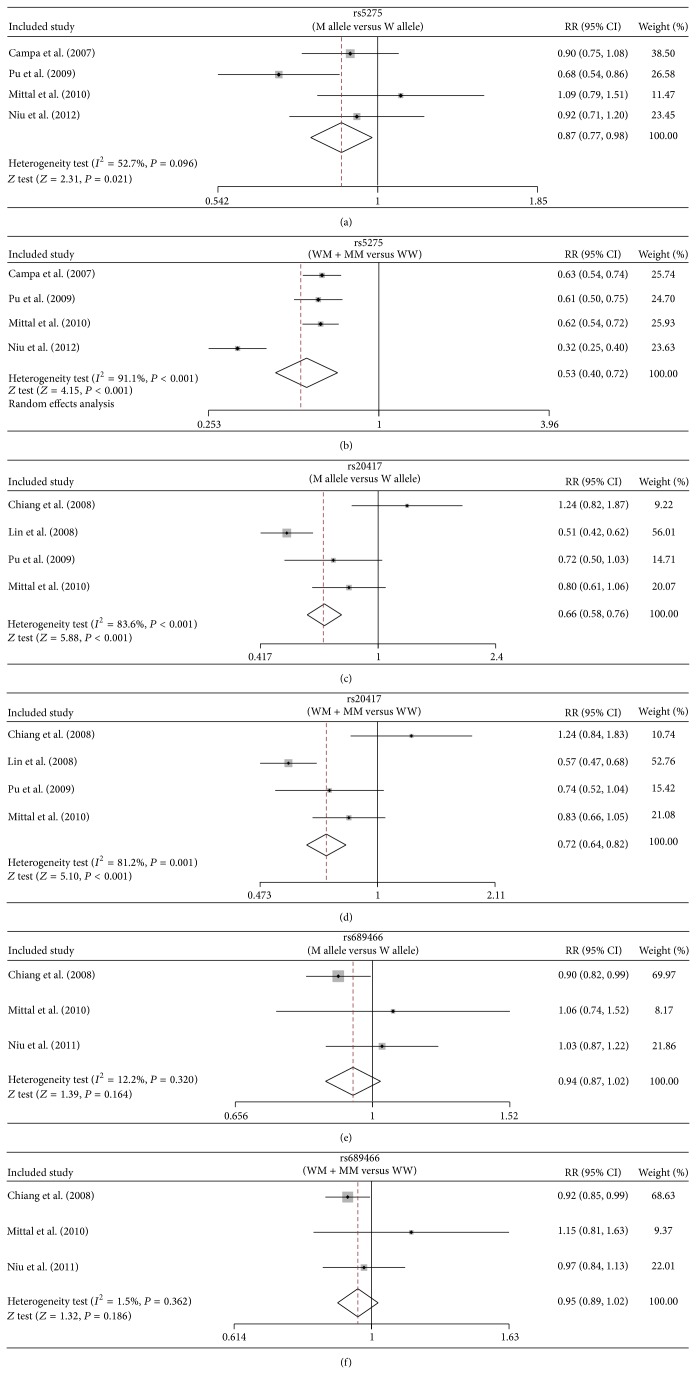
Forest analyses in present meta-analysis investigate the association between* COX-2* polymorphism and susceptibility to oral cancer ((a) +837 T > C (rs5275) in allele model; (b) +837 T > C (rs5275) in dominant model; (c) −765 G > C (rs20417) in allele model; (d) −765 G > C (rs20417) in dominant model; (e) 1195 A > G (rs689466) in allele model; (f) 1195 A > G (rs689466) in dominant model).

Four studies reported the association between +837 T > C (rs5275)* COX*-*2* gene polymorphism and the susceptibility to oral cancer. According to the heterogeneity test, the studies showed significant heterogeneity (allele model: *I* ^2^ = 52.7%, *P*_*h*_ = 0.096; dominant model: *I* ^2^ = 91.1%, *P*_*h*_ < 0.001). Our findings demonstrated that +837 T > C (rs5275) polymorphism in* COX-2* elevates the susceptibility to oral cancer and there were significant statistical differences in gene frequencies between the case and control groups in both allele model and dominant model (allele model: RR = 0.87, 95% CI = 0.77~0.98, *P* = 0.021; dominant model: RR = 0.53, 95% CI = 0.40~0.72, *P* < 0.001) (Figure 3, Table 2).

### 3.3. Meta-Analysis of Association between −765 G > C (rs20417) and the Susceptibility of Oral Cancer

A total of four studies reported that the −765 G > C (rs20417) polymorphism in* COX-2* related to the susceptibility to oral cancer. The result of the heterogeneity test indicated significant heterogeneity among various studies (allele model: *I* ^2^ = 83.6%, *P*_*h*_ < 0.001; dominant model: *I* ^2^ = 81.2%, *P*_*h*_ = 0.001). Our analysis suggested that the* COX-2* gene polymorphism 765 G > C (rs20417) is correlated with the susceptibility of oral cancers. The gene frequencies of case group and control group, under allele model and dominant model, exhibited significant differences (allele model: RR = 0.66, 95% CI = 0.58~0.76, *P* < 0.001; dominant model: RR = 0.72, 95% CI = 0.64~0.82, *P* < 0.001) (Figure 3, Table 2).

### 3.4. Meta-Analysis of Association between 1195 A > G (rs689466) and the Susceptibility of Oral Cancer

Three studies reported that the 1195 A > G (rs689466) polymorphism in* COX-2* linked with susceptibility to oral cancer. No significant heterogeneity was detected; thus fixed effects model was adopted (allele model: *I* ^2^ = 12.2%, *P*_*h*_ = 0.320; dominant model: *I* ^2^ = 1.5%, *P*_*h*_ = 0.362). Our findings of meta-analysis showed that 1195 A > G (rs689466) polymorphism in* COX-2* did not confer susceptibility to oral cancer. The gene frequencies of case group and control group under allele model and dominant model showed no significant differences (allele model: RR = 0.94, 95% CI = 0.87~1.02, *P* = 0.164; dominant model: RR = 0.95, 95% CI = 0.89~1.02, *P* = 0.186) (Figure 3, Table 3).

### 3.5. Sensitive Analysis and Publication Bias

**Figure 4:**
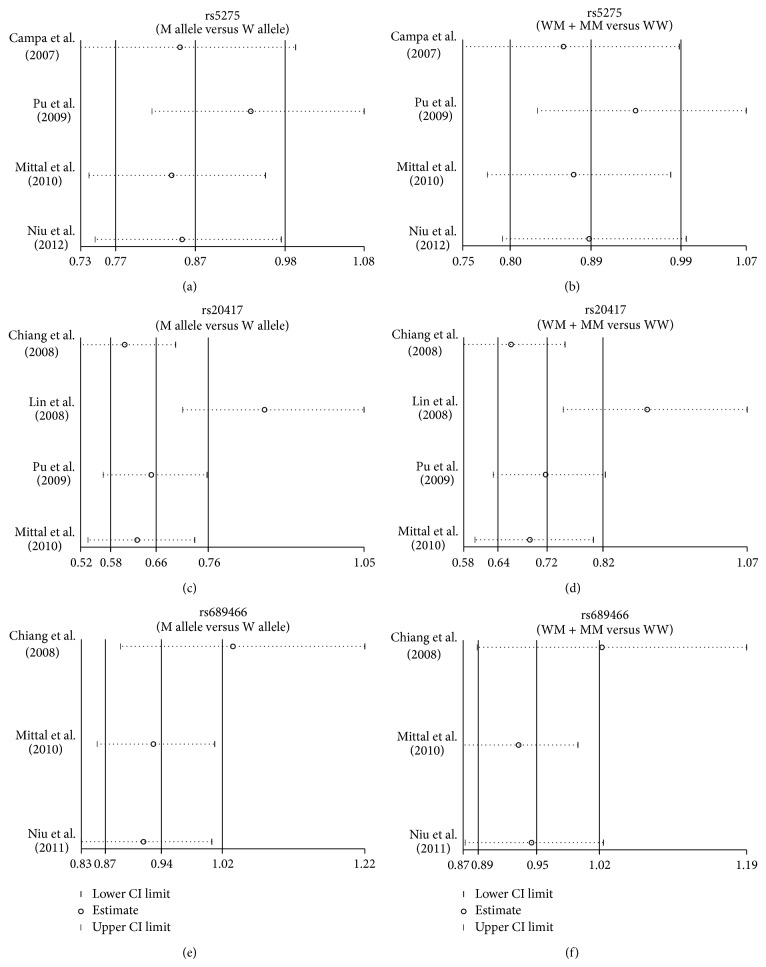
Sensitivity analysis in present meta-analysis investigates the association between* COX-2* polymorphisms and susceptibility to oral cancer ((a) +837 T > C (rs5275) in allele model; (b) +837 T > C (rs5275) in dominant model; (c) −765 G > C (rs20417) in allele model; (d) −765 G > C (rs20417) in dominant model; (e) 1195 A > G (rs689466) in allele model; (f) 1195 A > G (rs689466) in dominant model).

**Figure 5:**
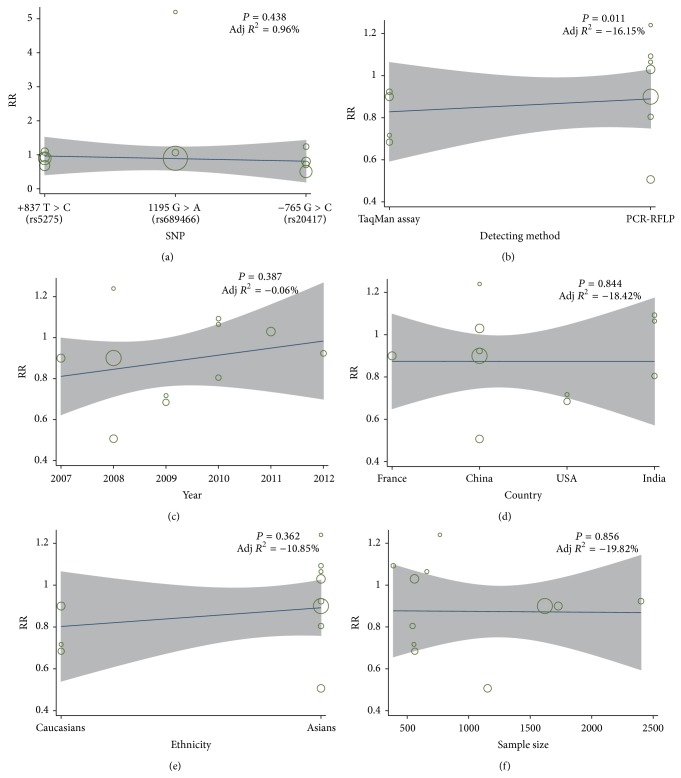
Metaregression analysis in present meta-analysis investigates association between* COX-2* polymorphisms and susceptibility to oral cancer ((a) +837 T > C (rs5275) in allele model; (b) +837 T > C (rs5275) in dominant model; (c) −765 G > C (rs20417) in allele model; (d) −765 G > C (rs20417) in dominant model; (e) 1195 A > G (rs689466) in allele model; (f) 1195 A > G (rs689466) in dominant model).

**Figure 6:**
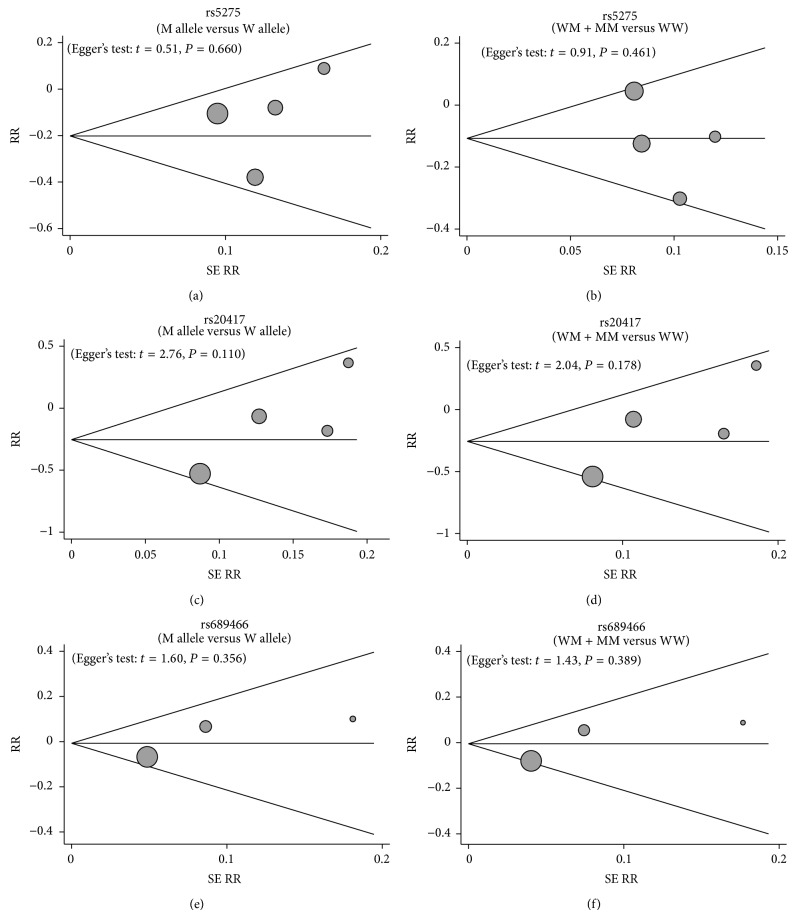
Funnel plot of publication in present meta-analysis investigates the association between* COX-2* polymorphisms and susceptibility to oral cancer ((a) +837 T > C (rs5275) in allele model; (b) +837 T > C (rs5275) in dominant model; (c) −765 G > C (rs20417) in allele model; (d) −765 G > C (rs20417) in dominant model; (e) 1195 A > G (rs689466) in allele model; (f) 1195 A > G (rs689466) in dominant model).

A sensitivity analysis indicated that, except for two selected studies, Lin (2008) related to −765 G > C (rs20417) and Chiang (2008) linked to 1195 A > G (rs689466); the remaining studies had no influence on the estimated pooled RR (Figure 4). The results of metaregression analysis suggested that SNPs, detecting method, year, country, ethnicity, and sample size were not the key factors for heterogeneity (Figure 5, Table 2). Funnel plots demonstrated no evidence of obvious asymmetry and Egger's test illustrated no presence of publication bias (*P* > 0.05), indicating highly reliable results (Figure 6).

## 4. Discussion

*COX-2* is an inducible enzyme that catalyzes the conversion of arachidonic acid to prostaglandins, and the reaction products influence cell proliferation and are key mediators of inflammation [9]. Evidences suggest that* COX-2* plays a key role in carcinogenesis by inhibiting apoptosis, promoting tumor growth, angiogenesis, invasion, and metastasis [16, 27–29]. Given the important roles of* COX-2* in the etiology of oral cancers, genetic variations of* COX-2* gene affect the susceptibility to cancer development [13]. In our meta-analysis, the* COX-2* +837 T > C (rs5275) and −765 G > C (rs20417) variant alleles were associated with significantly increased risk of oral cancer, while the effects of 1195 A > G (rs689466) need further exploration. The guanine (G) to cytosine (C) conversion at position −765 bp lies in the promoter region of* COX-2* gene, and the −765 G > C polymorphism affects transcription activity and is the most extensively studied* COX-2* polymorphism [11]. The −765 G > C (rs20417) located at the transcription start site prevents Sp1 binding but creates a new E2 promoter factor (E2F) binding site, leading to high transcription activity, which may be the mechanism underlying the increased cancer risk associated with −765 G > C (rs20417) polymorphism [30]. COX-2 promoter activity of −765C is reduced at 70% compared to −765G, and this change is associated with altered plasma levels of C-reactive protein, a marker for inflammation [23]. Furthermore, stability of* COX-2* mRNA is influenced by 3′UTR elements, and the exon 10 +837 T > C SNP is located in the 3′UTR and alters mRNA stability and translation efficiency to influence susceptibility to oral cancer [11, 14]. Thus, the two SNPs, +837 T > C (rs5275) and −765 G > C (rs20417), alter COX-2 protein levels by virtue of their effects on transcription and mRNA stability of COX-2 and modulate the degree and extent of inflammatory responses, contributing to individual variations in susceptibility to oral cancer [16]. Our conclusions are supported by previous observations that −765 G > C and +837 T > C polymorphisms are associated with high risk of oral cancer [10, 14].

Limitations of the present study should be acknowledged. First, the sample sizes in several of the incorporated studies were relatively small, which may reduce the strength of our conclusions. Second, all eligible studies were published in English and Chinese and indexed by the selected databases. It is possible that studies published in other languages or unpublished studies could be missed, which might bias the results. In addition, our result was on the basis of unadjusted estimates, while a more accurate analysis should be carried out if more detailed individual information was available, which would allow for an adjusted estimate by other causes such as age and sex.

In summary, our meta-analysis revealed a strong association between the −765 G > C and +837 T > C polymorphisms and the susceptibility to oral cancer. Therefore,* COX-2* polymorphisms, −765 G > C and +837 T > C, are linked to increased risk to oral cancers. However, the 1195 A > G polymorphism has no influence on oral cancer risk and will need to be explored further. More studies involving gene-gene and gene-environment interactions should also be taken into consideration in future analyses, which should lead to better, more comprehensive understanding of the correlation of the* COX-2* gene polymorphisms with the risk of oral cancers.

## Figures and Tables

**Table 1:** Baseline characteristics of the eligible studies for the relationship between functional *COX-2* gene polymorphisms and susceptibility to oral cancer.

First author	Year	Country	Ethnicity	Disease	Genotyping method	SNP
Campa [21]	2007	France	Caucasians	OSCC	TaqMan assay	+837 T > C (rs5275)
Chiang-a [22]	2008	Taiwan, China	Asians	OSCC	PCR-RFLP	1195 A > G (rs689466)
Chiang-b [22]	2008	Taiwan, China	Asians	OSCC	PCR-RFLP	−765 G > C (rs20417)
Lin [23]	2008	Taiwan, China	Asians	OSCC	PCR-RFLP	−765 G > C (rs20417)
Pu-a [26]	2009	USA	Caucasians	OPL	TaqMan assay	−765 G > C (rs20417)
Pu-b [26]	2009	USA	Caucasians	OPL	TaqMan assay	+837 T > C (rs5275)
Mittal-a [7]	2010	India	Asians	OSCC	PCR-RFLP	1195 A > G (rs689466)
Mittal-b [7]	2010	India	Asians	OSCC	PCR-RFLP	−765 G > C (rs20417)
Mittal-c [7]	2010	India	Asians	OSCC	PCR-RFLP	+837 T > C (rs5275)
Niu [24]	2011	China	Asians	OSCC	PCR-RFLP	1195 A > G (rs689466)
Niu [25]	2012	China	Asians	OSCC	TaqMan assay	+837 T > C (rs5275)

*Note*. OSCC: oral squamous cell carcinoma; OPL: oral precancerous lesions; PCR-RFLP: restriction fragment length polymorphism.

**Table 2:** Comparisons of genotype and allele frequencies between the case and the control groups for the relationship between functional *COX-2* gene polymorphisms and susceptibility to oral cancer.

SNP gene model	+837 T > C (rs5275)			−765 G > C (rs20417)			1195 G > A (rs689466)		
	RR	95% CI	*P*	RR	95% CI	*P *	RR	95% CI	*P *
M allele versus W allele (allele model)	0.87	0.77–0.98	0.021	0.66	0.58–0.76	<0.001	0.94	0.87–1.02	0.164
WM + MM versus WW (dominant model)	0.53	0.40–0.72	<0.001	0.72	0.64–0.82	<0.001	0.95	0.89–1.02	0.186
MM versus WW (homozygous model)	0.68	0.47–0.99	0.042	0.26	0.16–0.43	<0.001	0.85	0.71–1.01	0.069
MM versus WM (heterozygous model)	1.01	0.92–1.11	0.806	1.11	1.03–1.18	0.004	1.01	0.93–1.10	0.734
MM versus WW + WM (recessive model)	0.72	0.49–1.06	0.093	0.32	0.19–0.54	<0.001	0.91	0.73–1.12	0.363

RR: relative risk; 95% CI: 95% confidential intervals.

**Table 3:** Meta-regression analyses on SNP, detecting method, year, country, ethnicity and sample size for exploring potential source of heterogeneity between functional *COX-2* gene polymorphisms and susceptibility to oral cancer.

Heterogeneity factors	Coefficient	SE	*t*	*P* (adjusted)	95% CI	
					LL	UL
SNP	−0.071	0.082	−0.86	0.805	−0.299	0.158
Detecting method	1.964	0.812	2.42	0.181	−0.291	4.22
Year	0.301	0.130	2.32	0.206	−0.059	0.661
Country	0.124	0.098	1.27	0.588	−0.148	0.395
Ethnicity	−2.103	0.962	−2.19	0.232	−4.774	0.568
Sample size	0.002	0.001	2.21	0.230	−0.001	0.004

*Note*. SE: standard error; LL: lower limit; UL: upper limit; SNP: single-nucleotide polymorphism.
